# Femtosecond Laser Texturing of Surfaces for Tribological Applications

**DOI:** 10.3390/ma11050801

**Published:** 2018-05-15

**Authors:** Jörn Bonse, Sabrina V. Kirner, Michael Griepentrog, Dirk Spaltmann, Jörg Krüger

**Affiliations:** Bundesanstalt für Materialforschung und -Prüfung (BAM), Unter den Eichen 87, D-12205 Berlin, Germany; sabrina.kirner@bam.de (S.V.K.); michael.griepentrog@bam.de (M.G.); dirk.spaltmann@bam.de (D.S.); joerg.krueger@bam.de (J.K.)

**Keywords:** femtosecond laser processing, surface texture, laser-induced periodic surface structures, friction, wear, tribological tests, metals, steel, lubricants, oil, additives

## Abstract

Laser texturing is an emerging technology for generating surface functionalities on basis of optical, mechanical, or chemical properties. Taking benefit of laser sources with ultrashort (fs) pulse durations features outstanding precision of machining and negligible rims or burrs surrounding the laser-irradiation zone. Consequently, additional mechanical or chemical post-processing steps are usually not required for fs-laser surface texturing (fs-LST). This work aimed to provide a bridge between research in the field of tribology and laser materials processing. The paper reviews the current state-of-the-art in fs-LST, with a focus on the tribological performance (friction and wear) of specific self-organized surface structures (so-called ripples, grooves, and spikes) on steel and titanium alloys. On the titanium alloy, specific sickle-shaped hybrid micro-nanostructures were also observed and tribologically tested. Care is taken to identify accompanying effects affecting the materials hardness, superficial oxidation, nano- and microscale topographies, and the role of additives contained in lubricants, such as commercial engine oil.

## 1. Introduction

In a highly developed modern society, power generation, manufacturing, and transportation are central industrial activities—all relying on machines involving moving parts and interacting surfaces. For safe, reliable, and long-term employment of such machines, friction and wear on the interacting surfaces play an important role. The science and technology for investigating and understanding friction, wear, and lubrication of such interacting surfaces in relative motion are called *tribology* since 1966 [1]. Given its tremendous industrial and everyday-life relevance, during the following decades, the field of tribology has developed into an active field of research and development [2,3,4,5,6], with more than 500 scientific papers currently indexed per year with that term in the ISI Web of Knowledge database (status April 2018).

Recently, Holmberg and Erdemir analyzed the influence of tribology on the global energy consumption and the associated emissions and costs [7]. The authors estimated that, in total, about 23% of the world’s energy consumption and 8120 Mt/year of CO_2_-emission is caused by tribological contacts. Twenty percent of that is used to overcome friction and approximately 3% is spent to reconstruct worn parts or spare equipment. Globally, energy losses via friction and wear may potentially be reduced by 40% in the long term (15 years) and by 18% in the short term (8 years) upon developing and using new technologies for friction reduction and wear protection, e.g., in vehicles and in other equipment [7]. On a global scale, these savings are 1.4% of the Gross Domestic Product (GDP) annually and 8.7% of the total energy consumption in the long term—along with a reduction in CO_2_-emission globally by 3140 Mt CO2 [7]. From these arguments, it becomes immediately clear that the control of friction and wear has an enormous societal and economic relevance, which justifies exploring new and emerging technologies for improving tribological properties and potential applications. A strong demand can certainly be identified here for materials such as metals, which are especially used in industrial applications.

Since the surface topography and roughness have a remarkable impact on friction and wear, a promising approach for enhancing the tribological performance of surfaces is related to tailored surface texturing, which can be realized via laser processing. Properly designed grooves, protrusions, and dimples prepared on the micro- or nanoscale can have very beneficial effects. Moreover, the controlled flow of lubricants on a microscale may reduce friction and improve the load carrying capacity between sliding components. 

In this article, we first provide, in Section 2, a brief overview of the relevant definitions, the most common tribological test methods, and the basic ideas behind the (femtosecond) laser surface texturing mentioned in the previous paragraph. Section 3 summarizes original results obtained for a set of different “self-ordered” nano- and microstructures processed and tested under directly comparable experimental conditions. This extends the existing literature towards new surface morphologies and additionally supports the relevance of secondary chemical and structural effects influencing the tribological performance of the laser-textured surfaces.

## 2. Definitions, Methods, and Current State 

The following parts provide relevant definitions of tribological terms (Section 2.1) and test methods (Section 2.2), the specific ideas behind laser surface texturing for tribological applications (Section 2.3) and the particular benefits of using femtosecond lasers (Section 2.4). In all cases, the basic principles and methods are discussed briefly. For more details, the reader is referred to the specialized literature [2,3,4,5,6].

### 2.1. Definitions

*Friction* manifests itself as “consumption” of energy upon the relative motion of two bodies being in contact with each other, i.e., kinetic energy is usually converted into heat or other forms of energy. From the microscopic point of view, friction is caused by adhesion, deformation effects, and asperity removal of the surfaces involved [6]. Hence, the friction interaction is strongly affected by the intrinsic material structure of the bodies (e.g., hardness, determining which surface undergoes more wear/deformation), their surface topography (e.g., affecting the contact areas between them), and by chemical properties (e.g., affecting the surface wetting behavior or the tribological surface reactivity).

The *coefficient of friction* (COF), µ, is defined as the ratio of the friction force acting between two bodies in relative motion and the normal force (load) pressing them together. Thus, µ is a dimensionless scalar entity usually ranging from near zero to one. It can even reach values larger than this if, e.g., chemical bonds formed in the contact zone have to be overcome. The COF must be distinguished for static (µ_s_) and dynamic (kinetic, µ_k_) friction (typically µ_s_ > µ_k_). Opposed to common statements, the COF is not a material property, but rather a system property, as it is always related to a pair of materials pressed together or sliding against each other. It may also be affected by additional external influences, such as temperature, humidity of the atmosphere or potential presence of lubricants, specific test conditions (normal force, relative velocity, and geometry of the bodies in contact), surface contaminations, etc. COFs are typically measured experimentally by tribometers realizing specific test conditions (see Section 2.2).

*Wear* is the removal of material out of a contact zone on a solid surface generated by the action of another surface. For metals, wear typically occurs via the detachment of particles/the formation of wear debris, involving particle sizes ranging from atomic dimensions up to the millimeter range. In tribological applications, the wear rate typically changes with time or the number of sliding cycles through three stages: Stage I is the early “running-in” period, where the surface of the two bodies adapt to each other (involving significant wear rate variations); during this stage a tribological interlayer (“third body”) is often formed separating both materials. Stage II is a steady state regime, in which the operational life of the technical components is comprised. In the final Stage III, the components are subjected to failure due to an increased rate of wear.

*Lubricants*, such as oil, water, or other liquids, lower the mechanical shear stress, transport wear particles, and prevent their agglomeration and adhesion effects—all manifesting in a reduced COF. Lubricants for specific technical applications may contain particular *additives* for optimizing the viscosity, chemical stability, or even for providing beneficial tribological functionalities [4,8]. Even suitable solids (graphite, MoS_2_, etc.) exhibiting intrinsic crystallographic shear planes can act as lubricants or additives. For lubricated friction, the COF critically depends on the relative velocity of the sliding bodies. It is usually categorized through the Stribeck-curve in three different regimes [6], (i) *Boundary lubrication* (BL) at low velocities: The surfaces of the two solids directly come into contact, their load is carried mainly by asperities of the surfaces leading to a high COF; (ii) *Mixed lubrication* (ML) at medium velocities: The two solids have some asperity contact, their load is carried by surface asperities and the liquid lubricant leading to a reduction and a minimum of the COF; (iii) *Hydrodynamic lubrication* (HL) at high relative velocities: The two solids have negligible contact via asperities, their load is carried mainly by the liquid lubricant leading to a COF moderately increasing with the relative velocity. In this regime, the COF essentially depends on the viscosity of the lubricant.

### 2.2. Test Methods

The frictional properties of samples can be qualified by a *tribometer*. Such a device is a mechanical system, which brings two samples into contact under defined conditions. While the geometry of the surface may vary between the tribometer type as well as during the measurements, the tribometer may keep the ambient temperature/moisture stable; apply a load (normal force); control the relative motion of both surfaces (e.g., linearly reversing or continuous movement, velocities, etc.); record in-situ cycles, distances, and relative forces, which allow calculating the COF; and acquire the linear wear (depth of the wear tracks). The surfaces of the samples in contact adjust/deform due to the load applied so that it can be carried by the respective materials. Therefore, the pressure created in the contact zone by the load applied is of importance. This pressure strongly depends on the shape of the sample surfaces, i.e., the contact geometries. The actual/exact area of contact is hard to assess/determine. It is therefore intended to at least be able to calculate it for the starting conditions for reproducibility reasons. Hence, the shape of the test samples is kept as simple as possible.

Common tribological test (contact) geometries and methods are:

- Ball-on-disk (BoD): A fixed ball with a specified diameter is pressed against a flat sample surface; the relative motion can be realized by linearly reversing (reciprocating sliding), or by continuous or reversing rotation of the sample at a fixed distance between the tribological contact area and the rotation axis; test parameters are the load, the stroke (twice the amplitude), the reversing or rotational frequency, the number of test cycles or sliding distance, etc. [6].
- Pin-on-disk (PoD): The flat or curved surface of a fixed cylindrical pin with a specified diameter is pressed against a flat sample surface; relative motions and test parameters are the same as for the BoD contact geometry [5].
- Ring-on-disk (RoD): The flat surface of a ring is pressed and rotated against a flat sample surface; test parameters are the same as for the BoD contact geometry [6].
- Block-on-ring (BoR): A block sample is pressed against the curved outer surface of a rotating ring-shaped counterbody; test parameters are the same as for the BoD contact geometry [5].
- Scanning force microscope (SFM): Based on the mechanical contact between a nanometric sharp tip and a surface, the method allows to record a topography of the tested surface; in particular modes, it allows to image tribological properties, e.g., via lateral force measurements [9].
- There are also some other variants [5,6].

### 2.3. Basic Ideas behind the Laser Texturing for Tribological Applications

Laser processing has attracted a remarkable amount of research [10] for tailoring surface properties towards tribological applications [11]. This single-step technology allows a contactless treatment of almost arbitrary shaped workpieces with high precision. It can be applied precisely localized to submicrometric areas, but, via laser beam scanning, it is also feasible for large surface areas in the square meter range. In the context of tribology, laser processing is usually referred to as *Laser Surface Texturing* (LST). The ratio between the contact area and the area covered by the laser textured surface structures must often be carefully considered. Moreover, the elastic sample deformation during the mechanical load should not exceed the depths of the LST structures [12].

The following concepts were proposed to improve the tribological performance of surfaces via LST:

- Laser processing can be used to control the surface roughness via ablation.
- Laser-induced phase transitions, such as melting followed by rapid solidification, can modify the intrinsic material structure. This can increase the hardness of a near surface layer and improve wear resistance.
- Regular dimple, line, or grid patterns generated upon laser-processing at the surface may act as reservoirs for lubricants underneath the tribological contact area.
- Regular micro-dimple structures may generate a hydrodynamic pressure between oil-lubricated parallel sliding surfaces and act as a micro-hydrodynamic bearing, which allows enhancing the operational ranges [13,14,15].
- Laser-ablated microstructures may act as pockets for storing wear debris particles [16].
- Laser-processing with pulse durations in the µs- to ms-range can generate protruding microstructures, such as annular rims around the dimples or micro-welding dots for increasing the static COF (µ_s_) of metals [17].
- Laser-generated micro- and nanostructures may affect the surface wetting behavior, which can be relevant for lubricants [18,19].
- Laser-induced chemical reactions (such as oxidation in ambient air) may additionally affect the surface wetting behavior. Chemically altered surface layers may exhibit different mechanical properties or act as anchors for additive molecules contained in some lubricants.

Some of these concepts were already transferred to the automotive industry, for example the process of “laser-honing”, where laser structuring for reducing friction and wear is applied in the range of the upper piston reversal point of cylinders in combustion engines [20,21]. This additional manufacturing step is supposed to result in reduced oil and fuel consumption, less exhaust gas emissions, and an increased life-time of the engine. Etsion and Sher demonstrated in a commercial Diesel engine that laser surface textured piston rings reduced fuel consumption by 4 ± 0.5% with no traceable change in the exhaust gas composition or smoke level [21].

However, it must be noted here that a recent research project (2009–2013), founded by the German Federal Ministry of Education and Research, involving the key players of Germanys automotive industry could only partly confirm the beneficial tribological effects of dimples or line-shaped micro-pockets [22].

**Figure 1:**
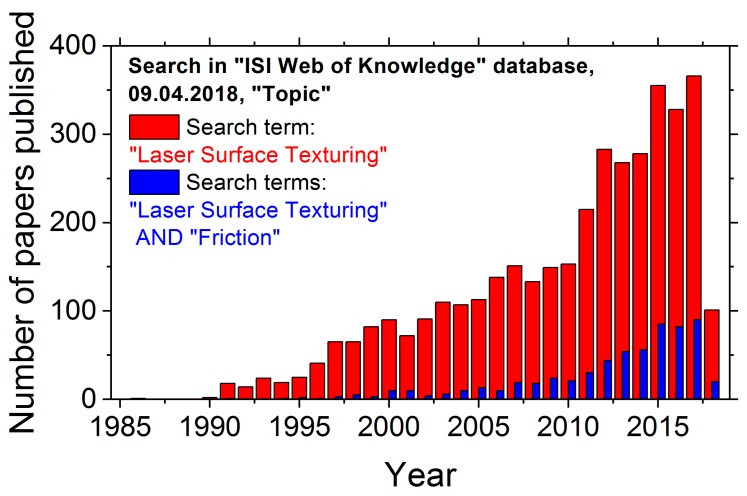
Research activities in the field of Laser Surface Texturing (LST), exemplified by the number of papers published per year—matching to the search term “Laser Surface Texturing” or to the two logically connected terms “Laser Surface Texturing” and “Friction” in the ISI Web of Knowledge database on 9 April 2018.

The trend of increasing research activities starting in the 1990s is visualized in Figure 1, which displays the number of peer-reviewed papers published per year in the field of LST (red bars). It currently indicates several hundreds of papers per year in this field. Moreover, an increasing number of publications among those is devoted to tribology (see the blue bars in Figure 1).

### 2.4. Femtosecond Laser Processing of Surfaces

Femtosecond (fs) lasers are an unprecedented tool for the processing of almost any material. This originates from the fact that their laser pulse duration is shorter than the electron–phonon coupling time, which is required to transfer the absorbed optical energy from the electron system of the material to its lattice (typically a few picoseconds). Consequently, the absorbed laser pulse energy stays widely confined during the laser–matter interaction and cannot proliferate via thermal diffusion to the surrounding of the irradiated region. Thus, the fs-laser features an increased precision for the ablation or modification of the processed material [23,24]. The high precision is accompanied by a minimized heat-affected zone (HAZ) extending a few hundred nanometers only [25]. This zone is by more than an order of magnitude smaller than the ones created via irradiation with ns- to ms-laser-pulses. Because of the improved precision and the reduced HAZ, rims or burrs surrounding the laser-irradiated regions are often negligible and additional mechanical or chemical post-processing of the sample surfaces is usually not required.

Different approaches were realized to apply femtosecond LST (fs-LST) for improving the tribological performance of surfaces. This includes the processing of:

- micrometer-sized dimples with optimized depths [15] or arranged in different geometries and distances [14];
- periodically spaced ablation line patterns with widths and periods in the range between a few and some hundreds of micrometers [14,26,27];
- periodic line patterns via two-beam-interference featuring periods typically between a few and some hundreds of micrometers—a technique frequently called *Direct Laser Interference Patterning* (DLIP) [28,29,30]; and
- *laser-induced periodic surface structures* (LIPSS, ripples) [31,32] with spatial periods in the sub-micrometer range, which form in a “self-ordered” way upon meandering laser-scan processing of surfaces.

LIPSS represent a widespread phenomenon which occurs upon treatment of solids by laser radiation [33]. For irradiation by linearly polarized ultrashort laser pulses, usually two different types of LIPSS are observed: so-called low-spatial frequency LIPSS (LSFL) having periods (Λ) close to the laser wavelength λ and high-spatial frequency LIPSS (HSFL) with periodicities significantly smaller than λ. For each type of LIPSS, the laser wavelength and the polarization are material-dependent key parameters for the spatial characteristics of the nanostructures, controlling their period and orientation. Additionally, the energy density (fluence, in J/cm^2^) and the number of pulses per spot can impose the type of LIPSS (HSFL or LSFL) and affect their spatial period. For metals, in most cases, LSFL with an orientation perpendicular to the laser beam polarization are observed [31,32]. These LSFL are generated by interference of the incident laser beam with a surface electromagnetic wave generated or scattered at the rough surface [32,34]. Moreover, it must be noted that usually a positive feedback mechanism is necessary for the formation of LIPSS. Upon repetitive laser pulse irradiations this mechanism selects specific spatial periods of the surface roughness distribution, which can absorb radiation best. Hence, the first pulses generate a rough surface that facilitates the coupling of energy for the subsequent laser pulses [35].

LIPSS are currently studied intensively as they allow the functionalization of surfaces towards the control of mechanical, chemical, or optical properties—featuring applications such as the “structural” colorization of technical surfaces [36,37], the control of surface wetting by different liquids [19,38,39], the mimicry of the natural texture of the integuments of animals [18,19,40], the tailoring of surface colonization by bacterial biofilms [41], etc. [42].

Almost 20 years ago, the potential of LIPSS for tribological applications was recognized: Yu and Lu reported an improved tribological performance of micrometer sized ripples on NiP (a support material for magnetic data storage elements in computer hard drives), when compared to the flat NiP surface [43]. In the following years, LIPSS were generated by ultrashort laser pulses on various materials and tested with respect to friction and wear, for instance for nitrides [44,45], carbon materials [46,47,48,49,50,51], semiconductors [52], and metals [12,53,54,55,56,57]. Several specific applications were explored, such as the improvement of mechanical seals [50,51], or the optimization of the machining performance of cutting tools [58,59,60,61].

In our previous work, we already studied various aspects of the tribological performance of femtosecond laser-induced periodic surface structures (fs-LIPSS) on different metals [12,32,42,53,54] and ceramics [45] under oil lubricated conditions. Those studies are extended here to other types of fs-laser-generated surface micro-structures, to a larger number of tribological tests cycles, and to dry test conditions. Moreover, imaging chemical analyses and local nanoindentation tests allow revealing the laser-induced chemical oxidation along with the presence of specific constituents of the additives contained in the engine oil, which are both acting in the tribological contact area. 

## 3. Results and Discussion

### 3.1. Self-Ordered Nano- and Microstructures

The following section provides an overview of the different fs-laser induced surface morphologies that are typically observed on strongly absorbing materials, such as metals. Apart from the LIPSS discussed already in the previous section, this also includes so-called *grooves* and *spikes* (also named cones) morphologies that form in a self-ordered (often termed self-organized) way and with particular spatial periods and orientations to the laser polarization under homogeneous laser irradiation.

#### 3.1.1. Surface Morphologies

Cylindrical slabs of hardened 100Cr6 steel (1.3505) were purchased from Optimol Instruments Prüftechnik GmbH (München, Germany) with 24 mm diameter, 8 mm height and a polished top-surface (average roughness *R*_a_ ≈ 35 nm). Commercial grade-5 titanium alloy (Ti6Al4V, 3.7165) was supplied by Schumacher Titan GmbH (Solingen, Germany) as rods of 25 mm diameter. These rods were cut into cylinders of 8 mm height. The mechanical polishing of their flat top-surface resulted in a mirror-like surface finish with an average roughness value *R*_a_ < 10 nm.

The fs-laser processing (center wavelength λ = 790 nm, pulse duration τ = 30 fs) was performed in scanning mode, where the sample was moved under the normally incident focused laser beam (Gaussian 1/*e*^2^ beam radius *w*_0_ ≈ 73 µm, linearly polarized) at a constant scan velocity *v*_x_ during the processing of a single line. The direction of the laser beam polarization was parallel to the scan direction. Considering the laser spot diameter *D* = 2*w*_0_, the effective number of laser pulses per focused spot can be calculated as *N*_eff_1D_ = (*D* × *f*)/*v*_x_ [62] from *v*_x_ and the laser pulse repetition frequency *f* (1 kHz). By scanning the focused laser beam in a meandering motion and with an inter-line offset Δ*y*, rectangular surface areas of several mm^2^ were processed by the fs-laser.

**Figure 2:**
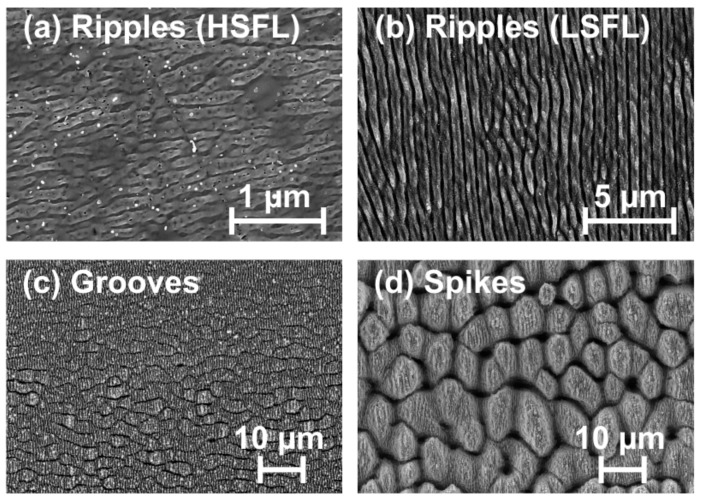
Top-view scanning electron micrographs of characteristic surface morphologies formed on 100Cr6 steel surfaces upon fs-laser area scanning (λ = 790 nm, τ = 30 fs, *f* = 1 kHz, line separation Δ*y* = 50 μm). Processing conditions: (**a**) HSFL: *φ*_0_ = 0.25 J/cm^2^, *N*_eff_1D_ = 20; (**b**) LSFL: *φ*_0_ = 0.5 J/cm^2^, *N*_eff_1D_ = 40; (**c**) Grooves: *φ*_0_ = 2.5 J/cm^2^, *N*_eff_1D_ = 100; and (**d**) Spikes: *φ*_0_ = 3 J/cm^2^, *N*_eff_1D_ = 400. The polarization direction is horizontal, which coincides with the scan direction. Note the different magnifications.

The dimensions of laser-induced surface structures essentially depend on the processing parameters peak fluence and the number of pulses per spot [32]. Upon fs-laser processing, *N*_eff_1D_ was systematically varied from 5 up to 1200, while the peak laser fluences (*φ*_0_) were chosen in the range between 0.15 and 3 J/cm^2^. By analyzing the laser-processed surfaces employing scanning electron microscopy (SEM, Carl Zeiss, Gemini Supra 40, Oberkochen, Germany), several characteristic surface morphologies were identified on 100Cr6 steel. Top-view SEM micrographs of four different characteristic surface morphologies are given in Figure 2 as examples, i.e., HSFL (Figure 2a), LSFL (Figure 2b), grooves (Figure 2c), and spikes (Figure 2d). The HSFL-ripples visualized in Figure 2a exhibit periods of 160 ± 60 nm only. LSFL-ripples with periods Λ of 620 ± 70 nm are depicted in Figure 2b, while grooves with distances of a few micrometers and spikes with typical lateral extents between 5 and 15 μm are shown in Figure 2c,d. The orientation of the ripples and the grooves depends on the direction of the laser beam polarization. LSFL are formed perpendicular to the laser beam polarization, while HSFL and grooves are generated parallel to it. In contrast, the spikes morphology is rather non-directional and represents a multi-scale structure: the micrometer-sized spike-like features are covered additionally by a nanoscale roughness, which also includes residuals of LSFL-ripples. Such hierarchical multi-scale structures exhibit a characteristic surface wetting behavior—as realized by nature through the well-known *Lotus-effect* (repelling water via hydrophobicity) [40]. These spikes structures are usually oleophilic on metals such as steel [19].

**Figure 3:**
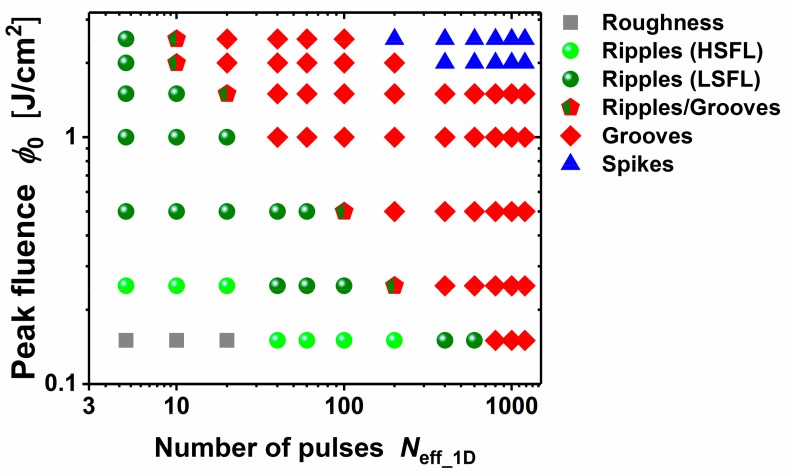
Morphological map of characteristic surface morphologies (ripples (HSFL and LSFL), grooves, and spikes) formed on 100Cr6 steel surfaces upon fs-laser area scanning at different irradiation parameters (processing conditions: λ = 790 nm, τ = 30 fs, and *f* = 1 kHz). Note the double logarithmical data representation.

Based on our experiments, a morphological map for area processing (Figure 3) was constructed for the 100Cr6 steel material. The map reveals that HSFL ripples are formed for a low effective number of laser pulses (*N*_eff_1D_ ≤ 200) and at peak fluences *φ*_0_ not exceeding ~0.25 J/cm^2^, i.e., close to the damage threshold of the steel. The LSFL-ripples are formed either at small *N*_eff_1D_ and fluences up to a few J/cm^2^ or for large *N*_eff_1D_ up to a few hundreds and at peak fluences below ~0.2 J/cm^2^. The spikes are generated for many pulses and at high fluences (*N*_eff_1D_ ≥ 200, *φ*_0_ ≥ 2 J/cm^2^). The grooves cover the parameter range bordered by the LSFL-ripples and the spike structures.

#### 3.1.2. Tribological Performance

Reciprocating sliding tribological tests (RSTT) were performed with an inhouse-built tribometer [63] (BoD geometry, see Section 2.2). The samples were tested at a frequency of 1 Hz, with a stroke of 1 mm, and with a normal force of *F*_N_ = 1.0 N against a polished and hardened ball made of 100Cr6 steel (10 mm diameter, *R*_a_ ≈ 6 nm average roughness) as counterbody (regime of mixed friction).

Using a Hertzian elastic deformation model of a sphere in contact with a flat sample, the radius of the ball–sample contact area was estimated [64]. At the given normal force, the contact radius lies between 30 and 40 µm for both materials tested. Hence, it is about 2–3 orders of magnitude larger than the LIPSS periods. Moreover, at the conditions given, the Hertzian model predicts material (hardness) dependent sample–ball deformations between 200 and 300 nm along with maximum contact pressures of several hundred MPa. These deformation values are of the order of the typical modulation depths of the LSFL and smaller than the topographic height modulations of grooves and spikes.

In the RSTT experiments, 1000–100,000 cycles were performed in a synthetic paraffin oil (free of additives) as well as in a fully formulated engine oil with additives (Castrol VP-1). The experimental uncertainty range in the COF measurements is ±0.02. To remove the residual lubricants, the samples were cleaned after the tribological tests for five minutes in an ultrasonic bath with petroleum ether.

**Figure 4:**
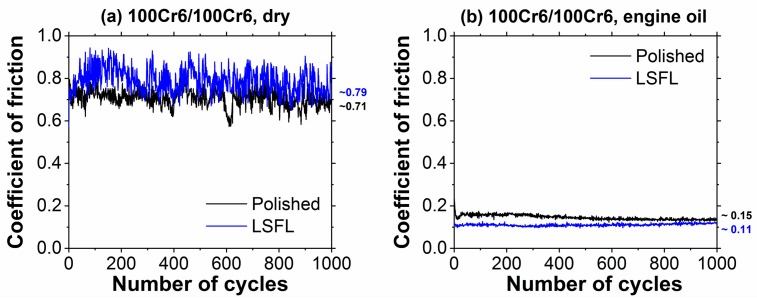
Coefficient of friction as a function of the number of cycles as obtained during reciprocating sliding tests (RSTT: normal force 1.0 N, stroke 1 mm, frequency 1 Hz, cycles 1000) of the polished (black) and of the fs-laser structured (LSFL-covered) 100Cr6 surface (blue) against a 100Cr6 10-mm steel ball: (**a**) dry; and (**b**) in engine oil (Castrol VP-1). The numbers at the end of the curves represent the mean values averaged over 1000 sliding cycles.

Figure 4 compares the COF measured during 1000 RSTT cycles for dry sliding at room temperature (Figure 4a) and for lubricated sliding in engine oil (Figure 4b) for both a polished reference (black curves) and for the fs-laser processed (LSFL covered) 100Cr6 samples (blue curves). Under dry conditions, the COF of the LSFL-covered surface is slightly increased compared to that of the polished reference, as manifested in average COF values of 0.79 (LSFL) and 0.71 (polished), respectively.

In engine oil, the COF is significantly reduced to values clearly below 0.15 (Figure 4b). Here, the fs-laser structured (LSFL-covered) surface exhibits an average COF around ~0.11 being reduced by ~24% when compared to the polished surface finish (COF ~0.15). Although being close to the experimental uncertainty, the friction reduction is in line with other studies [55,56,57], which reported a beneficial effect of the LIPSS on steel in various lubricants/environments and under somewhat different tribological conditions.

**Figure 5:**
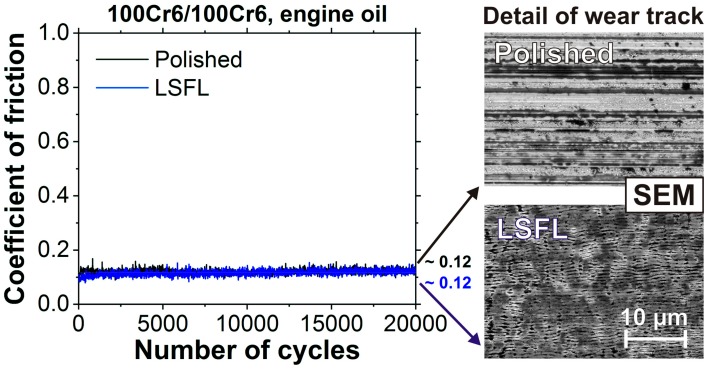
Coefficient of friction as a function of number of cycles as obtained during reciprocating sliding tests (RSTT: normal force 1.0 N, stroke 1 mm, frequency 1 Hz, cycles 20,000) of the polished (black) and of the fs-laser structured (LSFL-covered) 100Cr6 surface (blue) against a 100Cr6 10-mm steel ball in engine oil (Castrol VP-1). The numbers at the end of the curves represent the mean values averaged over 20,000 sliding cycles.

Since in Figure 4b the two curves of the LSFL-covered and the polished surface were slowly approaching each other, tribological test was repeated with a 20-times larger number of sliding cycles at fresh test sites. The results are compiled in Figure 5. In the left part of the figure, the COF is shown as a function of the number of sliding cycles, while, on the right, high-resolution scanning electron micrographs are presented, taken close to the center of the wear tracks. Although the COF is almost identical around ~0.12 after a few thousand of sliding cycles up to 20,000 cycles, the SEM images clearly reveal that the LSFL have mostly survived the RSTT and are not completely worn out. Hence, we conclude that LSFL on 100Cr6 steel may moderately decrease the COF during the first few thousands of sliding cycles in engine oil.

Table 1 summarizes the tribological performance of the different self-ordered micro-/nanostructures (LSFL-LIPSS, Grooves, and Spikes) processed by fs-laser irradiation on 100Cr6 steel surfaces. The left column provides the average COF for dry sliding, while the right column presents the corresponding data for sliding in engine oil.

**Figure 6:**
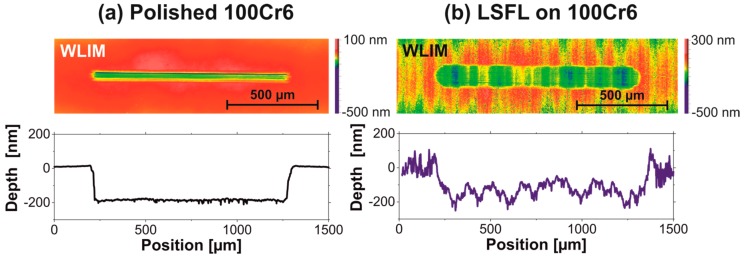
White light interference microscopic topographies of the wear tracks shown in Figure 5 along with cross-sectional line profiles parallel to the RSTT sliding direction: (**a**) wear track after 20,000 sliding cycles on the polished reference surface; and (**b**) wear track on the fs-laser structured (LSFL-covered) surface after 20,000 sliding cycles.

To quantify the change of the surface topography, white light interference microscopy (WLIM) was performed (Zygo, NexView, Middlefield, OH, USA) imaging the wear tracks on 100Cr6 samples after the tribological tests as shown in Figure 5. The surface topographies of the corresponding wear tracks are provided in the upper row in Figure 6a for the polished reference surface and in Figure 6b for the fs-laser processed (LSFL-covered) surfaces. Note that, at the magnification selected, the WLIM cannot resolve the LIPSS topography here. However, a surface topography modulation caused by the line-wise laser processing can clearly be seen in Figure 6b. The vertical modulation accounts to less than 200 nm here. The lower row in Figure 6 represents horizontal cross-sections through the deepest regions of the wear tracks. 

The maximum depth of the wear tracks is rather similar here (~200 nm), although, on average, the track at the polished surface is shallower than the wear track in the LSFL-covered region. However, a remarkable difference can be seen in the width of the two wear tracks, indicating that the surface mechanical properties may have changed upon the fs-laser processing.

**Figure 7:**
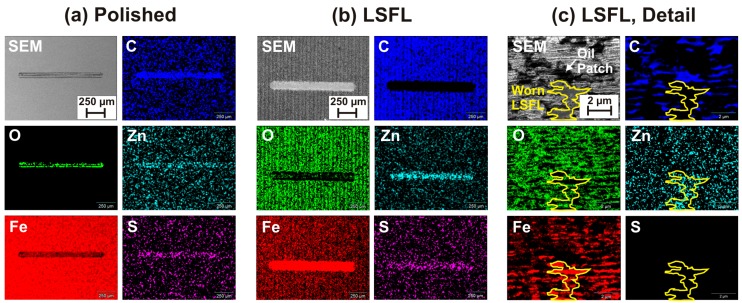
SEM micrographs and corresponding EDX maps of the spatial distributions of the elements O, Fe, C, Zn, and S in the wear tracks associated with the COF measurements shown in Figure 5 (20,000 sliding cycles): (**a**) overview of the wear track on the polished surface; (**b**) overview of the wear track on the LSFL-covered laser processed surface; and (**c**) magnified detail within the wear track shown in (**b**) (EDX: (**a**,**b**) 5 kV; and (**c**) 3 kV)).

To reveal chemical alterations caused by the fs-laser processing or by the tribological tests, the wear tracks shown in Figure 6 were analyzed by energy dispersive X-ray analysis (EDX, Thermo Fisher Scientific, Thermo NSS 3.1, Waltham, MA, USA). Chemical elements related to the sample material (Fe, O, and C), or associated with the engine oil (C, Zn, and S) were mapped in and around the wear tracks. The elemental maps are assembled in Figure 7 along with corresponding SEM micrographs. The left and the middle column visualize the elemental distributions of the entire wear tracks after 20,000 sliding cycles on the polished (Figure 7a) and on the fs-laser processed (LSFL-covered) 100Cr6 steel surface (Figure 7b). More detailed high-resolution elemental maps acquired within the laser-processed wear track are provided in the right column (Figure 7c).

The wear track on the polished 100Cr6 steel surface (Figure 7a) exhibits increased signals of O, C, Zn, and S, while the signal of Fe is reduced. This is indicative of the presence of a tribo-layer formed during the RSTT, which contains the elements of the additive zinc-dialkyl-dithiophosphate (ZDDP, Zn[S_2_P(OR)_2_]_2_, with R representing an alkyl chain [8]), which is included in the engine oil and of the oil itself (C). The tribo-layer covers the surface within the wear track and, thus, reduces the Fe-signal of the bulk sample material.

In contrast, the wear track on the fs-laser processed (LSFL-covered) 100Cr6 steel surface (Figure 7b) shows increased signals of Fe, Zn, and S and a reduced signal of C. The oxygen-signal is reduced compared to the fs-laser processed area around the wear track. However, in the center of the wear track a local increase is seen. Those elemental distributions indicate that a fs-laser-induced oxide layer is formed, which may additionally contain carbon. Upon RSTT, the laser-oxidized layer is partly removed, which explains the reduced oxygen- and carbon-signal and the increased Fe-signal. In a central part of the wear track, the signal of Zn, O, and S are locally increased, indicating again the formation of a tribo-layer (as in the wear track generated at the polished surface).

Additional information can be obtained by high-resolution elemental maps acquired in the central part of the wear track in the fs-laser processed zone (Figure 7c). The electron acceleration voltage was reduced to 3 kV here to probe the chemistry in a more surface near layer. In the corresponding SEM image, two characteristic regions are marked, i.e., a characteristic dark region (labeled “Oil patch”) and a zone where the LSFL have been partly removed during the RSTT. The latter is framed by a yellow line (labeled “Worn LSFL”) and indicated in all corresponding elemental maps. In regions where the LSFL were partly polished away, the Fe-signal is increased and the oxygen signal is strongly reduced. In the valleys of the LSFL topography being not affected by the RSTT, the opposite behavior is evident. The C-signal is very pronounced in the regions appearing black in the SEM image supporting the interpretation that these dark patches are caused by residuals of the engine oil, which were not completely removed in the ultrasonic bath cleaning after the RSTT. Within the Worn LSFL region, the C-signal is very low, while Zn is clearly increased.

The EDX data strongly suggest that a thin tribo-layer was formed at the tribologically tested surface regions, while the WLIM data indicate that the surface mechanical properties may have changed upon the fs-laser processing. Hence, another RSTT experiment with 100,000 sliding cycles (and all other parameters kept as in Figure 5) was performed in the fs-laser treated (LSFL-covered) region to reinforce the superficial modifications. The COF was almost constant at the level (average COF ~0.13 ± 0.01, curve not shown here for brevity), very similar to the data shown in Figure 5. The corresponding wear track was subjected to instrumented indentation testing (IIT), allowing to measure the local hardness of the material at a selected indentation depth. For that, a Keysight G200 Nanoindenter system was used, equipped with a DCM II measuring head. The measurements were performed according to the ISO 14577 standard [65]. Using the so-called continuous stiffness method (CSM), a small oscillatory force was applied to the indenter during its continuous loading to a certain depth limit. For materials exhibiting plastic-elastic behavior, the dynamic response was used for continuously measuring the dynamic stiffness of the contact region as a function of both, the indentation depth and the frequency. Based on the dynamic stiffness data, the indentation hardness *H*_IT_ and indentation modulus *E*_IT_ were calculated. The experimental parameters are: depth limit 500 nm, amplitude and frequency of displacement oscillation 1 nm and 75 Hz.

The indentation hardness *H*_IT_ was averaged over a depth range between 100 and 200 nm, which is typical for the initial plastic deformation in our tribological tests. Single indentations (each separated by 5 µm) were performed along a line crossing perpendicular through the center of the wear track in the fs-laser processed (LSFL-covered) region. For comparison, the same procedure was repeated in a fs-laser processed (LSFL-covered) region without a wear track and at the polished reference surfaces.

**Figure 8:**
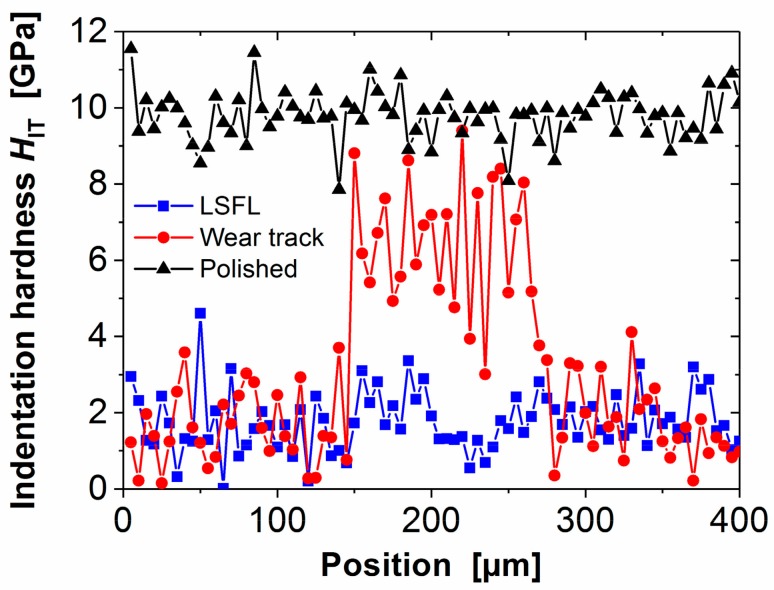
Indentation hardness *H*_IT_ across the 100Cr6 steel surface, tested in three different regions: polished (black triangles), fs-laser processed (LSFL-covered, blue squares), and across the center of an RSTT wear track (RSTT: normal force 1.0 N, stroke 1 mm, frequency 1 Hz, cycles 100,000, Castrol VP-1) in the fs-laser-processed (LSFL-covered, red circles) area.

Figure 8 compiles the results of the indentation hardness *H*_IT_ as function of the lateral position for all three measurements, i.e., along lines crossing the wear-track (red circles), on the LSFL-covered region (blue squares), and on the non-irradiated (polished) region (black triangles). A remarkable difference of the indentation hardness can be seen between the polished 100Cr6 steel surface (*H*_IT_ ~8–12 GPa) and the fs-laser processed one (*H*_IT_ < 4.5 GPa), confirming that the fs-laser processing reduces the hardness of the material in a surface near region [66]. Consequently, the measurements related to the wear track show the same low indentation hardness outside the track on the LSFL-covered region. Within the track, the hardness is increased to a level between that of the polished and of the laser-processed materials (*H*_IT_ ~3–9 GPa). The width of ~120 µm of locally increased *H*_IT_ coincides with the width obtained from the SEM images of the wear track. Hence, it is obvious that, during the RSTT, the material softened upon the fs-laser treatment is partly removed, uncovering the harder material underneath.

### 3.2. Hybrid Micro-Nanostructures

#### 3.2.1. Surface Morphologies

**Figure 9:**
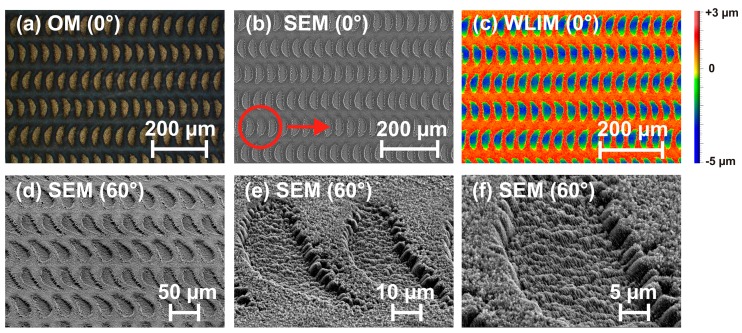
Top-view: (**a**) optical micrograph; (**b**) SEM micrograph; and (**c**) WLIM topography of hybrid micro-nanostructures processed on Ti6Al4V-alloy (*φ*_0_ = 0.25 J/cm^2^; *N*_eff_1D_ = 600, λ = 790 nm, τ = 30 fs, *f* = 1 kHz, *w*_0_ ≈ 73 µm, line separation Δ*y* = 90 μm, area 7 × 7 mm^2^). The red circle in (**b**) indicates the Gaussian beam diameter *D* for comparison. The red arrow marks the direction of line scanning which is parallel to the laser beam polarization. The lower row (**d**–**f**) displays detailed magnifications taken under 60° by SEM.

Upon exploring the different surface morphologies that can be generated on titanium-alloy (Ti6Al4V) upon fs-laser processing—in analogy to the results presented in Section 3.1.1 for 100Cr6 steel, some novel sickle-shaped dimple structures covered by LSFL-ripples were found. These hybrid micro-nanostructures are visualized in Figure 9 via top-view optical microscopy (OM, Nikon, Eclipse L200, Tokyo, Japan, Figure 9a), top-view scanning electron microscopy (SEM, Figure 9b) and white light interference microscopy (WLIM, Figure 9c). Tilted-view detailed SEM magnifications are complemented in Figure 9d–f.

These structures are formed on Ti6Al4V at fluences of *φ*_0_ = 0.25 J/cm^2^ and for a large pulse overlap (*N*_eff_1D_ = 600), i.e., in the ablative regime. At first sight, the sickle-shaped features seem to be an imprint of an imperfect laser beam profile. However, the latter can be ruled out here since the orientation of the sickles turns 180° if the sample movement direction is reversed in the next line during the meandering processing. In the case of an imperfect beam profile, all sickle-shaped structures would exhibit the same orientation regardless of the direction of the scanning. Since thermal accumulation effects cannot play a role here at 1 kHz pulse repetition rate, we speculate that an interplay of interpulse oxidation, material redeposition, and local absorption causes these specific hybrid micro-nanostructures. In such a scenario, the spatially rising edge of the scanned Gaussian-like beam profile promotes the superficial oxidation of the Ti-alloy surface. As a result of the large pulse overlap of 99.8% (*N*_eff_1D_ = 600), the thickness of the oxide layer gradually increases until it acts as a reflectivity reducing coating. Since then less pulse energy is reflected, the absorbed energy density (per volume) increases and, upon exposure to several pulses and local fluences close to the maximum, may generate ablation at the chemically modified surface in a sickle-shaped region. The trailing (low fluence) edge of the scanned laser beam subsequently again oxidizes the surface. The WLIM measurements indicate a lateral size of about 30 × 60 µm^2^ and a maximum depth of ~4.5 µm (Figure 9c).

Note that irregular structures during continuous line scanning at constant velocities were observed also for fs-laser waveguide writing in dielectrics employing high repetition rate lasers. In contrast to the present case, the irregularity is caused by transiently changing thermo-physical properties/absorption upon heat accumulation (see [67] and references therein), modulating the locally absorbed optical energy along the scanned line. Moreover, it should be mentioned that the sickle-shaped structures were not observed on steel surfaces under similar laser processing conditions. This further supports the proposed oxidation scenario since titanium shows an extraordinary affinity to oxygen.

#### 3.2.2. Tribological Performance

**Figure 10:**
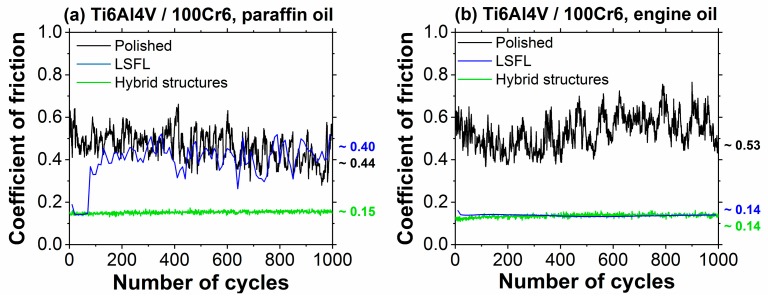
Coefficient of friction as a function of the number of cycles as obtained during reciprocating sliding tests (RSTT: normal force 1.0 N, stroke 1 mm, frequency 1 Hz, 1000 cycles) of the polished (black) and of the fs-laser structured (hybrid structures covered: green, LSFL-covered: blue) Ti6Al4V surface against a 10-mm 100Cr6 steel ball in: (**a**) paraffin oil; and (**b**) an engine oil (Castrol VP-1). The data for the LSFL-covered surfaces are taken from Ref. [53] and were acquired with 10-times less data points. The numbers at the end of the wear tracks represent the mean values averaged over all acquired data points.

Figure 10 displays as green curves the COF of the hybrid micro-nanostructures as measured during 1000 RSTT sliding cycles of a 100Cr6 steel ball in paraffin oil (Figure 10a) and in engine oil (Figure 10b). Corresponding reference curves for the polished surface finish are provided as black curves. For comparison with a previous publication [53], the data for LSFL-covered surfaces are complemented as blue curves.

**Figure 11:**
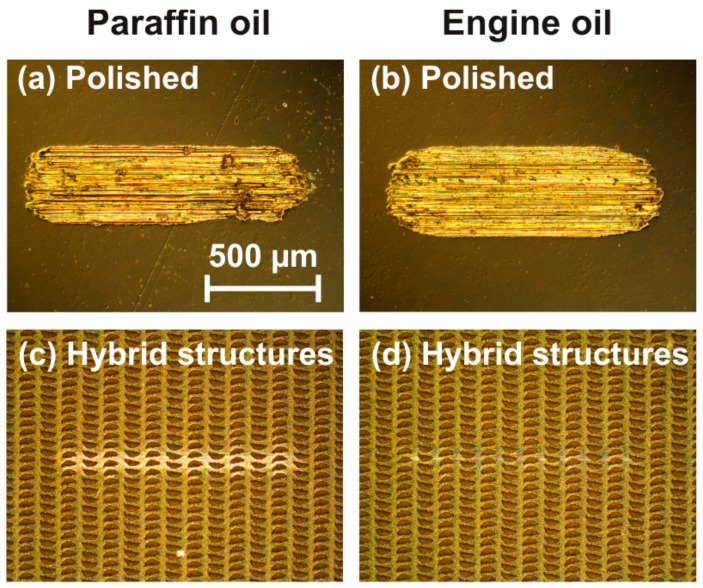
Optical micrographs (differential interference contrast) of the wear tracks on differently conditioned Ti6Al4V surfaces associated with the COF measurements shown in Figure 10: (**a**,**c**) RSTT with 1000 sliding cycles in paraffin oil; and (**b**,**d**) RSTT with 1000 sliding cycles in engine oil (Castrol VP-1). A common scale bar is provided in (**a**).

In both lubricants, the hybrid micro-nanostructures exhibit low average COFs which are almost constant around ~0.15 (paraffin oil) and ~0.14 (engine oil) over the entire range of 1000 sliding cycles. In contrast, the polished reference surfaces exhibit significantly larger average COFs (paraffin oil: ~0.44; engine oil ~0.53) along with strong variations. Both are indicative that the polished surface was severely damaged during the RSTT—as supported by the corresponding optical micrographs provided in Figure 11a,b.

While the COF of the LSFL-covered surface tested in engine oil is at a very similar level (~0.14, see Figure 10b) as that of the hybrid micro-nanostructures over the entire range of sliding cycles, the COF of the LSFL-covered surface tested in paraffin oil starts at a similar low level, but increases significantly after ~100 running-in cycles (Figure 10a). The different behavior of the LSFL-covered surfaces in both lubricants was attributed to the presence of the additive (ZDDP) contained in the engine oil but not being present in the paraffin oil along with the laser-induced surface modifications [53].

**Figure 12:**
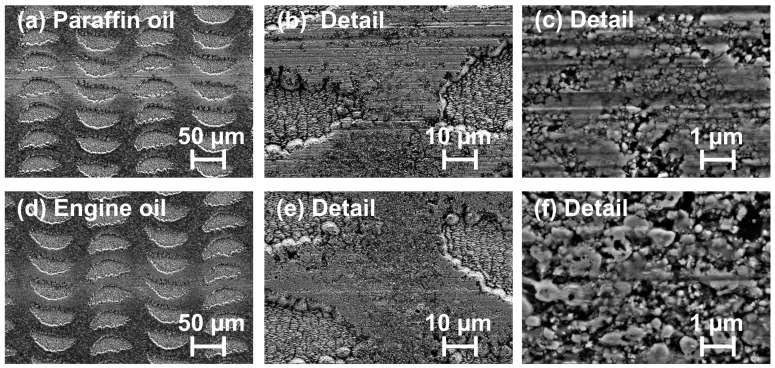
(**a**–**c**) Top-view SEM micrographs of the wear track shown in Figure 11c after sliding in paraffin oil; and (**d**–**f**) top-view SEM micrographs of the wear track shown in Figure 11d after sliding in engine oil (Castrol VP-1).

The fact that the hybrid micro-nanostructures exhibit a low COF over the entire range of sliding cycles even in the paraffin oil indicates some new mechanisms acting here. The optical micrographs shown in Figure 11c,d confirm that the laser-structured surface stayed widely intact during the RSTT, pointing towards somewhat less wear in engine oil compared to paraffin oil. The latter becomes even clearer from the collage of scanning electron micrographs of both wear tracks (paraffin oil vs. engine oil) provided in Figure 12. Moreover, it is interesting to note that the surface reflectivity is locally increased within the wear tracks (see Figure 11)—as expected from the hypothesized sickle-formation scenario upon a partial removal of the reflectivity reducing laser-induced oxide layer in the tribological test.

Regarding the beneficial tribological performance of the fs-laser processed sickle-shaped hybrid micro-nanostructures on Ti6Al4V-alloy, two aspects should be underlined. (i) The sickle-shaped dimple patterns can act as a reservoir for the lubricants confining it in the tribological contact area; (ii) The irradiation conditions for these structures (*φ*_0_ = 0.25 J/cm^2^; *N*_eff_1D_ = 600) supposedly generate a thicker oxide layer than that used for the generation of the LSFL (*φ*_0_ = 0.11 J/cm^2^; *N*_eff_1D_ = 56). As shown in recent publications, this laser-induced oxide layer supports the reduction of friction and wear in ZDDP-containing engine oil if a certain layer thickness is exceeded [66,68].

The following conclusions can be drawn from the results presented in Section 3 regarding the tribological performance of different self-ordered micro- and nanostructures tested by RSTT: (1) fs-laser processing can generate characteristic self-ordered surface textures on various metals (steel and titanium alloys), i.e., LIPSS, grooves, and spikes. (2) While during the RSTT running-in stage (1000 cycles) under dry testing conditions, the LSFL-ripples on 100Cr6 steel exhibit an increased COF compared to the flat polished reference surface, the COF is reduced by ~24% in a commercial engine oil containing the additive ZDDP. This reduction of the COF diminishes for a larger number of test cycles (i.e., 100,000 cycles), then approaching the COF value of the reference. (3) Chemical analysis by EDX revealed that the fs-laser texturing is accompanied by a superficial oxidation, which lowers the material hardness. For Ti6Al4V titanium alloy, the presence of the laser-induced oxide layer along with the additive ZDDP is beneficial for reducing friction and wear, as demonstrated before for the LSFL-ripples and confirmed here also for some new hybrid micro-nanostructures.

## 4. Future Perspectives

Femtosecond lasers are versatile tools, which are currently entering industrial production. Taking benefit of their ultrashort pulse durations, even below the time scales of thermal heat flows, allows promoting matter into extreme conditions. This enables the processing of materials with high lateral precision featuring surface structures in the sub-100 nm range. Given the scalability of the treated areas by proper choice of laser parameters and manufacturing strategies, the fs-laser processing can be adapted to almost all industrial demands. Apart from an enhanced control of the surface topography, the fs-laser irradiation will even allow tailoring the local surface chemistry. The combination of micro-nanostructures (driving capillary effects) and the surface wetting properties (contact angles of desired liquids) will allow controlling the motion and the flow of lubricants in a tribologically beneficial way. In combination with a suitable choice of the processing environment, this technology may enable numerous future tribological applications.

## Figures and Tables

**Table 1:** Coefficient of Friction, COF (µ_k_), averaged over 1,000 cycles (RSTT, 1 mm, 1 Hz, 1 N, RT) in different environments (dry; engine oil Castrol VP-1: 100Cr6 vs. 100Cr6).

Surface	COF µ_k_ (Dry)	COF µ_k_ (Engine Oil)
Non-irradiated	0.71	0.15
LSFL ^1^	0.79	0.11
Grooves ^1^	0.73	0.13
Spikes ^1^	0.70	0.15

^1^ Stroke perpendicular to the laser processed lines.
